# Evolutionary Genetics of Microbial Symbiosis

**DOI:** 10.3390/genes12030327

**Published:** 2021-02-25

**Authors:** Laura Baldo, John H. Werren

**Affiliations:** 1 Department of Evolutionary Biology, Ecology and Environmental Sciences, University of Barcelona, 08028 Barcelona, Spain; 2 Institute for Research on Biodiversity (IRBio), University of Barcelona, 08028 Barcelona, Spain; 3 Department of Biology, University of Rochester, Rochester, NY 14627, USA

## Introduction

Symbiosis is the living together of dissimilar organisms [1,2]. As such, symbiotic relationships can range from antagonist (parasitic) to mutualistic and can vary along this continuum within and between species in space and time. Microbial symbioses encompass a wide array of players (e.g., bacteria, fungi, and small eukaryotes) and are an integral part of organismal life, contributing to phenotypes at all levels of biological organization, from molecules to ecosystems.

There has been an explosion of microbiological research on symbiosis emerging from the omics revolution, which has made many previously intractable symbioses available for dissection. Current research ranges from unraveling the biochemical interactions among symbiont partners to uncovering the incredible ecological diversity and dynamics of microbial communities and host associations. Host-microbes engage in extensive and complex cross-kingdom molecular dialogue, where symbionts can modulate their reciprocal gene expression patterns, complement metabolic pathways, and combine genetic information through DNA exchange, in some cases becoming sufficiently integrated through coinheritance to be considered as an evolutionary unit of selection. This extensive genetic and biochemical interplay has enormous implications in the emergence of novel traits and the overall diversification of life.

In this Special Issue, we present a collection of new reviews and original research papers that target the genetic machinery behind the evolution of microbial symbioses, using both classical and novel experimental model systems. These include microbial symbioses with arthropod hosts [3,4,5], leguminous plants [6,7], social amoebae [8], and the largely understudied symbiosis of fungi in insects [9]. These papers provide key examples of current research on the microbial genetic repertoire (genes, genomic features, and their molecular evolution) and metabolic interactions (including biochemical and cellular mechanisms) involved in symbiotic interplay.

Among the most widespread and abundant symbionts are the obligate intracellular bacteria *Wolbachia* [10]. Here Lindsey (2020) reviews the molecular basis of *Wolbachia*-host interactions by examining the bacterial genes involved in sensing, responding to, and modifying the host environment [3]. By inquiring 29 *Wolbachia* genomes, Lindsey found evidence for a small but largely conserved set of genes coding for transcriptional factors, proteins involved in two-component sensing-response systems, and two types of bacterial secretion systems. The secretion machinery likely allows *Wolbachia* to colonize an extraordinary variety of hosts and facilitates the manipulation of host physiology in many ways. Lindsey finally presents an overview of current studies on gene-regulation in *Wolbachia*, stressing the need for more in-depth bioinformatic analyses coupled with experimental essays that can directly target the regulatory pathways involved in the diverse array of *Wolbachia*-host interactions.

Chen et al. (2020) provide a compelling review of genetic and biochemical aspects of the most common reproductive manipulation induced by *Wolbachia* in insects, cytoplasmic incompatibility (CI) [5]. This is a phenotype where uninfected eggs fail to develop if fertilized by sperm from infected males or when males and females harbor *Wolbachia* with different compatibility types. After decades of research on the topic, recent studies have finally pointed to a pair of syntenic operons encoding CI-inducing factors (Cifs) as key players in CI-induction and rescue. The two different operons, which can act independently to cause CI, encode Cif proteins with distinct enzyme activities. Most Cifs are derived from a viral prophage, suggesting a trans kingdom gene transfer at the origin of this widespread phenotype. Chen et al. (2020) present current knowledge on the biochemistry of these newly discovered Cifs and the cell biological mechanisms that take place during CI to evaluate mechanistic models that can explain this decades-old mysterious reproductive manipulation.

Among facultative symbionts are the widespread *Burkholderia*. These environmentally acquired bacteria are incredibly diverse in their host range and in the phenotypes that they induce (from mutualism to pathogenesis). Here Takeshita and Kikuchi (2020) and Miller et al. (2020) explore the nature of the *Burkholderia* symbiosis in insects [4] and amoebae [8], respectively. Takeshita and Kikuchi present a genomic study on the bean bug symbiosis with *Burkholderia* bacteria, which are environmentally acquired and harbored in the host midgut. This is a promising new model for experimental manipulation of the host-bacteria interactions. By applying the organ culture method, they succeeded in isolating and sequencing the genomes of two novel strains of newly discovered *Paraburkholderia* symbionts from the plant bug *Physopelta gutta*. Their comparative genomic analysis highlights genes and functions conserved with other stink bug-associated *Burkholderia*, which may be essential for gut adaptation in insects.

Miller et al. (2020) explored infection patterns of *Paraburkholderia* symbionts in the social amoeba *Dictyostelium discoideum*, a powerful experimental model to study the mechanism of bacterial infection and pathogenesis [8,11]. By exposing the amoebae to several bacterial strains of three *Paraburkholderia* species, they found that an increase in bacterial infection titer is not detrimental to host spore productivity (i.e., it does not appear to impose a large fitness cost). The authors suggest that the bacteria’s ability to establish a high infection load without compromising host fitness is a key selective advantage for the symbiont. They highlight the need to explore the interplay between infection patterns and host outcomes for a comprehensive understanding of the evolutionary trajectories of symbiosis.

Among classical models of symbiosis are the root nodule bacteria (rhizobia) in leguminous plants. Kimeklis et al. (2019) and Chirak et al. (2019) present of pair of sister articles exploring the genetic diversity of the symbiotic bacteria *Rhizobium leguminosarum* in a relict legume plant, *Vavilovia formosas*, the closest putative living relative of the last common ancestor of the Fabeae tribe [6,7]. Kimeklis et al. (2019) performed a comparative genetic analysis of *Rhizobium* symbiotic (*sym*) housekeeping genes and showed that the *Vavilovia* isolates form a separate group within *R. leguminosarum* as a consequence of the long-term ecological isolation and the ancestral phylogenetic position of the host plant [6]. Chirak et al. (2019) investigated details of the microevolutionary variation of *sym* gene arrangements in *Vavilovia* symbionts and found an important genomic reorganization indicative of intensive bacterial evolution in the legume nodules [7].

Finally, this special issue contains a comprehensive review by Martinson (2020) on understudied fungus-insect symbioses [9]. Here Martinson proposes to revive an early historical model of fungal symbiosis between fungi in the genus *Symbiotaphrina* and Anobiid beetles. This system offers a compelling example of the mutualistic relationship between gut fungi and insects, in which the fungus provides the host with essential vitamins. The manageability of the system, where both host and fungus can be grown separately, and, as the fungus has both an intra and extracellular stage, offers great potential for experimental manipulations aimed at dissecting the genetic and functional aspects of this very ancient association.

Overall, this set of papers represents an important resource on both classic and emerging microbial symbioses systems, illustrating how advances in genomics and molecular biology can be used to reveal the intricacies of these intimate relationships.
